# Predicting norovirus and rotavirus resurgence in the United States following the COVID-19 pandemic: a mathematical modelling study

**DOI:** 10.1186/s12879-023-08224-w

**Published:** 2023-04-20

**Authors:** Brooke L. Lappe, Mary E. Wikswo, Anita K. Kambhampati, Sara A. Mirza, Jacqueline E. Tate, Alicia N. M. Kraay, Ben A. Lopman

**Affiliations:** 1 Gangarosa Department of Environmental Health, Rollins School of Public Health, Emory University, 1518 Clifton Rd, Atlanta, GA 30322 USA; 2 Division of Viral Diseases, Centers for Disease Control and Prevention, Atlanta, GA USA; 3 Department of Kinesiology and Community Health, University of Illinois at Urbana-Champaign, Champaign, IL USA; 4 Epidemiology Department, Rollins School of Public Health, Emory University, Atlanta, GA USA

**Keywords:** Norovirus, Rotavirus, Transmission, Seasonality, Mathematical modelling, Surveillance, COVID-19

## Abstract

**Background:**

To reduce the burden from the COVID-19 pandemic in the United States, federal and state local governments implemented restrictions such as limitations on gatherings, restaurant dining, and travel, and recommended non-pharmaceutical interventions including physical distancing, mask-wearing, surface disinfection, and increased hand hygiene. Resulting behavioral changes impacted other infectious diseases including enteropathogens such as norovirus and rotavirus, which had fairly regular seasonal patterns prior to the COVID-19 pandemic. The study objective was to project future incidence of norovirus and rotavirus gastroenteritis as contacts resumed and other NPIs are relaxed.

**Methods:**

We fitted compartmental mathematical models to pre-pandemic U.S. surveillance data (2012–2019) for norovirus and rotavirus using maximum likelihood estimation. Then, we projected incidence for 2022–2030 under scenarios where the number of contacts a person has per day varies from70%, 80%, 90%, and full resumption (100%) of pre-pandemic levels.

**Results:**

We found that the population susceptibility to both viruses increased between March 2020 and November 2021. The 70–90% contact resumption scenarios led to lower incidence than observed pre-pandemic for both viruses. However, we found a greater than two-fold increase in community incidence relative to the pre-pandemic period under the 100% contact scenarios for both viruses. With rotavirus, for which population immunity is driven partially by vaccination, patterns settled into a new steady state quickly in 2022 under the 70–90% scenarios. For norovirus, for which immunity is relatively short-lasting and only acquired through infection, surged under the 100% contact scenario projection.

**Conclusions:**

These results, which quantify the consequences of population susceptibility build-up, can help public health agencies prepare for potential resurgence of enteric viruses.

**Supplementary Information:**

The online version contains supplementary material available at 10.1186/s12879-023-08224-w.

## Background

The COVID-19 pandemic, caused by severe acute respiratory syndrome coronavirus 2 (SARS-CoV-2), began in the United States in January 2020. A variety of national and state level non-pharmaceutical interventions (NPIs) such as social distancing, mask wearing, and improved hand hygiene were implemented to curb SARS-CoV-2 incidence and hospitalizations. Federal social distancing guidelines were announced March 16, 2020, and included limiting gatherings to fewer than 10 people, avoiding eating and drinking in bars and restaurants, and avoiding unnecessary travel. These federal guidelines remained in effect through the end of April 2020 [1]. From then, social distancing guidelines and lockdowns were managed by state or local authorities and varied across the nation. Social contacts were reduced as a result of guidelines and personal behavior changes during the initial mitigation period between February and May 2020. A review of social contact patterns during the pandemic found that most studies reported a mean of between 2 and 5 contacts per person per day, a substantial reduction compared to pre-COVID-19 rates, which ranged from 7 to 26 contacts per day [2, 3].

These NPIs also reduced the transmission of enteric pathogens, with a > 80% in incidence of reported norovirus outbreaks in the United States from April to July 2020 [4, 5]. This reduction in reports was consistent across many pathogens and observed in countries where NPIs for the control of SARS-CoV-2 have been implemented [6, 7]. Later, introduction of vaccinations and subsequent reduction in COVID-19 hospitalizations and deaths in the United States led to contact patterns increasing but still not reaching pre-pandemic levels [8].

Norovirus is an endemic enteric virus with no current vaccination or specific treatment options. Globally, one out of every five cases of acute gastroenteritis that leads to diarrhea and vomiting is caused by norovirus [9]. Norovirus is a seasonal virus with annual recurring peaks in incidence during the wintertime (typically October to February). Norovirus affects all ages and is generally mild but in more vulnerable populations can cause hospitalization or death [10]. Repeat infections are common throughout childhood and adulthood, as a result of relatively short-term immunity [11]. Norovirus is transmitted through direct contact with infected people or contaminated surfaces and consumption of contaminated water and food [10]. Norovirus can spread easily and quickly, and the majority of infections are spread via person-to-person contact, so changes in contact patterns can influence norovirus transmission [12].

Rotavirus is the leading cause of severe diarrhea, hospitalization, and diarrhea- related deaths globally in infants and children < 5 years old [13]. Prior to the introduction of rotavirus vaccines in the U.S., there were an estimated 2.7 million cases per year [14]. Since the introduction of vaccination in 2006, rotavirus incidence has declined between 57%-89% [14, 15]. Rotavirus is also a winter-spring seasonal virus, but vaccination has shifted it from a recurring annual virus to a biennial virus. Rotavirus immunity dynamics are dependent on vaccination rates. Rotavirus is transmitted through the fecal–oral route and is contact dependent, with most exposure occurring person to person [10]. Rotavirus mostly affects children < 5 years old, and transmission to older family contacts is low (< 25%) [10]. While rotavirus can cause repeat infections, subsequent illnesses are generally not severe, and rotavirus is predominantly considered a childhood illness [16].

We aimed to assess changes in population susceptibility and community incidence of norovirus and rotavirus gastroenteritis episodes that resulted from the COVID-19 pandemic, and project the future trends in incidence as contacts resume and other NPIs are relaxed. We developed pathogen-specific transmission models to reflect their natural history and, for rotavirus, vaccination, and fitted the models to surveillance data up to December 2019. We then made predictions for trends in norovirus and rotavirus disease trends until 2030 under different scenarios of contact patterns informed by mobility data.

## Methods

Our overall analytic approach was to develop pathogen-specific models for norovirus and rotavirus, to fit those models using likelihood to Centers for Disease Control and Prevention (CDC) surveillance data using time-varying contact rates, and then to project the fitted models into the future under different scenarios of increasing contacts. Two separate models were developed to capture the differences in natural history of norovirus and rotavirus and vaccination (in the case of rotavirus). For both models, U.S. birth rates were informed by the CDC Wonder database [17]. We assumed death rates equaled birth rates so the total population remained constant [18, 19]. We also included seasonal forcing of the transmission probability. Full model diagram and equations for rotavirus are also defined in the supplemental appendix (Supplemental Fig. 1). All modeling and analysis were done in R (version 4.0.4).

**Fig. 1:**
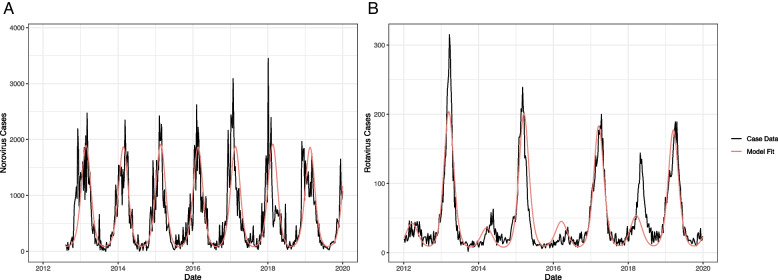
Model fit for (**A**) norovirus (SIRS model) cases from August 2012–December 2019 and (**B**) rotavirus (SIRV model) cases from January 2012–December 2019

### Data for model fitting

We fitted the models to a weekly time series of norovirus outbreak-cases and rotavirus case reports to CDC. Cases were used for model fitting and the model with the best fit based on maximum likelihood was used for prediction by forward simulation. For norovirus, the total number of norovirus outbreak-associated cases occurring August 2012–December 2019 and reported to the National Outbreak Reporting System (NORS) by states participating in the Norovirus Sentinel Testing and Tracking network (NoroSTAT). NORS is a web-based platform used by states to report data on all foodborne, waterborne, and enteric disease outbreaks spread by person-to-person or animal contact, contact with contaminated environmental surfaces, or by an unknown mode of transmission. The twelve states participating in NoroSTAT report all epidemiologically suspected and laboratory-confirmed norovirus outbreaks to NORS within 7 business days of identification and provide minimum outbreak data elements including the setting and number of cases for each outbreak [20, 21]. Data were extracted from NORS on November 22, 2021.

For rotavirus we used data on rotavirus cases reported to the National Respiratory and Enteric Virus Surveillance System (NREVSS) during January 2012–December 2019. Laboratories participating in NREVSS voluntarily report to CDC the total aggregate number of weekly tests performed and number of positive tests for various pathogens, including rotavirus. We used the number of aggregate positive tests to indicate the number of cases. Data were extracted on November 22, 2021.

### Norovirus

A deterministic susceptible-infected-recovered-susceptible (SIRS) model was developed for norovirus, drawing on our previous studies [11, 22, 23] (Supplemental Fig. 1). To represent the population of the states participating in NoroSTAT, we modeled a population of 60 million, informed by US Census data of the 12 states [24]. Susceptible individuals were infected at a rate $\lambda$. Individuals were assumed to move from the susceptible to the infectious class where they stayed for an average of 12 days, based on a weighted average of the duration of the infectious asymptomatic, symptomatic, and post-symptomatic periods [22, 25, 26]. Upon moving to the recovered class, individuals were assumed to have waning immunity to further symptomatic infection for 5.1 years^13^, at which point individuals moved from the recovered class back to the susceptible class, denoted as rate ω [22].

### Rotavirus

A deterministic susceptible-infected-recovered-vaccinated (SIRV) model was developed for rotavirus in the United States population under 5 years, since it primarily a childhood illness, transmission to adult contacts is low, and repeat infections in adulthood are generally not severe (Supplemental Fig. 1). The population was calculated as the average population under 5 from 2010–2019, approximately 23.97 million [24]. We assumed individuals were vaccinated at birth at a coverage (ρ) of 77% and vaccine efficacy (χ) of 90% [27]. Susceptible individuals were infected at a rate $\lambda$ and entered the infectious class. Upon infection, individuals were assumed to move from the susceptible to the infectious class where they stayed for an average of 5 days [28]. For simplicity, waning immunity was omitted such that individuals remained in the recovered class until death. While immunity to infection for rotavirus does wane, subsequent infections generally occur at lower rates and are less severe [29, 30]. We considered this an appropriate simplification since we modeled reported cases, which are generally severe and most likely to be primary infections of young children [16, 31, 32].

We estimated transmission parameters (*q*), seasonality parameters (*β*_*1*_*, w*), and reported fraction (*r*) for norovirus and rotavirus models using maximum likelihood estimation. The calculated parameters that minimized the negative-log-likelihood were used for forward projections.

### Time varying contact patterns

The average number of daily contacts prior to the pandemic was assumed to be 13.94 for both models [33]. From February 4, 2020 and onwards, we assumed the average daily contacts was reduced by the percent reduction in mobility, informed by Institute for Health Metrics and Evaluation (IHME) mobility estimates for the U.S. The mobility estimates were used to parameterize models of the time-varying contacts which were reduced as a result of the pandemic. We calculated mobility estimates as the average percent reduction in mobility on a scale from 0 (no mobility reduction) to 1 (100% mobility reduction) from February 4, 2020. After April 5, 2020, when the maximum percent reduction of 53% was reached, the mobility reduction percentage was multiplied by the average number of contacts per individual. After that maximum percent reduction, we increased contacts to varying scenarios in which adults returned to 70%, 80%, 90%, or 100% (“return to normal”) of their pre-pandemic contact rates (Supplemental Fig. 2).

**Fig. 2:**
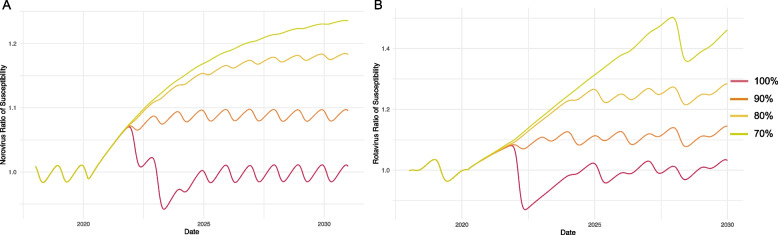
Ratio of susceptibility calculated as a change relative to pre-pandemic averages for norovirus (**A**) and rotavirus (**B**) and predictions for 70–100% contact resumption scenarios

## Results

From April 2020 (federal stay-at-home order) to April 2021, there was a substantial reduction in norovirus cases (90.4% compared to the April 2019-April 2020 time period) and rotavirus cases (81.2% compared to the April 2018-April 2019 time period) in the NORS and NREVSS surveillance data we analyzed. This period corresponds to a reduction in mobility ranging from 18–53%.

### Model fitting

The maximum likelihood models of norovirus and rotavirus captured key features including seasonality, post-vaccine biennial pattern for rotavirus, and reasonable parameter estimates (e.g., transmission probability, seasonal amplitude and offset, and reported fraction) (Table 1). The model fit for norovirus cases and rotavirus cases is shown in Fig. 1.

**Table 1:** Norovirus SIRS model and rotavirus SIRV model parameter value and sources

		**Norovirus**		**Rotavirus**	
**Parameter**	**Symbol**	**Value**	**Sources**	**Value**	**Sources (Citation)**
Initial Susceptible	*S*_*init*_	60,000,000 people	US Census Data	23,970,000 people	US Census Data
Infectious Period	**σ**	12 days	Weighted average	5 days	Heymann, 2015 [26]
Duration of immunity	*ω*	5.1 years	Lopman et al. 2014 [20]	Lifelong	Assumption
Average number of contacts per person	*c*	14 /day	Mossong et al. 2008 [32]	14/day	Mossong et al. 2008 [32]
Probability of transmission per infectious contact	$q$	0.0078	Fitted	0.397	Fitted
Seasonal amplitude	*β*_*1*_	0.3	Fitted	0.08	Fitted
Seasonal offset	*w*	4.1	Fitted	0.02	Fitted
Reported fraction	*r*	0.011	Fitted	0.029	Fitted
Vaccine efficacy	χ	Not applicable		0.9	Jonesteller et al. 2017 [17]
Proportion vaccinated	ρ	Not applicable		0.77	CDC [27]

### Population susceptibility

We calculated a ratio of the population susceptible relative to the pre-pandemic average. According to the best-fit model, the population susceptibility began to change around August 2020, when it began to increase and peaked in October 2021 at 1.07, representing a 7% increase from the pre-pandemic year because of waning immunity and decreased infections. Following the population susceptibility high of 1.07, the population susceptibility was projected to dip to 0.94 in December 2022 and then reach pre-pandemic equilibrium in 2024 (Fig. 2).

The population susceptibility for rotavirus was lower than for norovirus due to vaccination, lack of waning immunity in the model, and a higher reproductive number (based on the transmission probability). Our modeled population susceptibility increased and peaked at a high of 1.06 in November 2021. Following November 2021, the population susceptibility was projected to dip at 0.87 in May 2022 (Fig. 2).

### Predicted impacts following a resumption of contacts

**Fig. 3:**
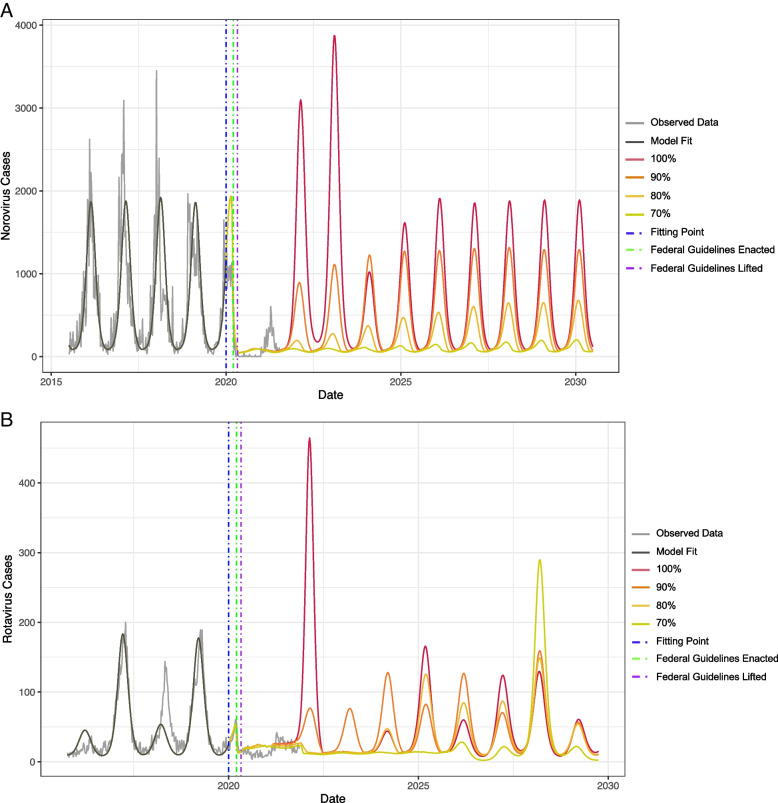
Data used for model fitting and model estimates of predictions of norovirus (**A**) and rotavirus (**B**) from 2015–2030, compared with a typical pre-pandemic modeled year

With the exception of the 100% “return to normal” scenario, predicted norovirus cases never exceeded pre-pandemic levels, reaching their highest levels for at 7%, 25% and 68% in year 2025 for the 70, 80 and 90% resumption scenarios, respectively (Fig. 3). In the scenario where contacts resumed to 100% pre-pandemic levels, the number of norovirus cases was predicted in 2022 to be 175% higher, and in 2023 to be 167% higher compared to a pre-pandemic year.

Predictions for rotavirus activity were similar and predicted cases never exceeded pre-pandemic levels, reaching their highest levels at 8%, 65% and 59% of pre-pandemic activity in year 2025 for the 70%, 80%, and 90% resumption scenarios, respectively (Fig. 3). The biennial pattern of rotavirus was disrupted by the COVID-19 pandemic and changes in contact patterns. In the 100% “return to normal” scenario, the number of cases was predicted to increase in 2022 by 360% and decrease in 2023 by 78% (compared to the pre-pandemic biennial pattern, in which 2022 would be a low-incidence year and 2023 would be a high-incidence year). Cases were predicted to remain relatively low after this 2022 surge until 2025.

## Discussion

Our results showed a two-fold increase in case incidence for both viruses under the 100% “return to normal” scenario. Based on our modeling, this increase in community incidence persisted for two years for norovirus, and one year for rotavirus. Our model quantified the consequences of build-up of susceptible population over the pandemic and suggests the impact on norovirus and rotavirus transmission dynamics would last for several years, which has clinical and public health consequences. By 2026, norovirus transmission returned to its pre-pandemic equilibrium under the 100% scenario, but this was not the case for rotavirus, which did not reach pre-pandemic equilibrium under the 100% scenario for the entire projection through 2030. The differences in disease burden and time to equilibrium could be explained by the different transmission drivers for norovirus and rotavirus. For norovirus, immunity is short-lasting, only acquired through infection, and partial immune escape driven by evolving new variants is possible. For rotavirus, population immunity is driven partially by vaccination, and as a result, the younger age groups drive immunity more than older age groups. Changes in mobility patterns compared to pre-pandemic levels reflect how people changed behaviors during the course of the COVID-19 pandemic and can thus serve as a time-varying partial proxy for broader societal disruptions related to the pandemic. This increase in susceptibility to infection reflects declining population immunity and, therefore, risk of resurgence with resumption of contacts. For instance, the large projected peak in rotavirus cases under the 70% contact resumption scenario is a result of a long period of population susceptibility build-up and finally resurging. Our results found that changes in contact patterns affect short- and long-term incidence of disease. Rotavirus biennial disease dynamics are particularly sensitive to changes in contact patterns.

There are several limitations of this study. We assumed a SIRS model for norovirus and a SIRV model for rotavirus, which is an oversimplification for those pathogens. This analysis was completed prior to 2022, so surveillance data for 2022 was not incorporated into the modeling. NORS surveillance data for 2022 has found a resurgence in norovirus outbreaks, but it does not exceed pre-pandemic levels. This could be a result of contacts not truly reaching their pre-pandemic levels because of permanent behavioral changes from COVID-19 waves, such as hand-hygiene stations remaining in public places or people continuing to work from home. We used mobility data as a proxy for contacts, which does not address the complexity of masking, social distancing, working from home, etc. The predictions are sensitive to assumptions about contacts and the probability of transmission, which remains uncertain. It could also be a result of our model not completely capturing immunity correctly, including differing tiers such as by age or include incremental immunity. For norovirus particularly, we did not capture genotype replacement even though it is a driver of norovirus population persistence. Our parameter estimates were limited in complexity, insensitive to changes in the environment, and only represented a single point in time. The transmission probability, or *q*, was particularly sensitive to randomness that is not captured in our SIRS and SIRV models. For norovirus, we used outbreak-associated cases for norovirus, which does not account for sporadic cases. Additionally, not all norovirus transmission occurs via direct person-to-person contact [10] and our model does not explicitly account for foodborne or waterborne transmission, although it could implicitly capture these transmission sources by using the overall transmission rate. For rotavirus, we used lab testing of sporadic cases, which may not capture outbreak-associated cases. Additionally, we did not capture decreases in vaccination rates for children during the pandemic, which some studies estimate decreased as much as 7% [34]. This is of particular concern for rotavirus because of a strict vaccination schedule which makes catch-up vaccination impossible. As a result, our results may underestimate case resurges for rotavirus given transmission dynamics are so sensitive to vaccination.

The clinical and public health importance of this study is that our findings suggest that there may be an increased risk of norovirus and rotavirus over the coming years due to a reduction in contact rates that occurred during the COVID-19 pandemic. Our models suggest that norovirus and rotavirus case incidence is likely to substantially increase beyond what has been experienced in years prior to 2020 if contact rate is to resume to 100%. The lower incidence of norovirus and rotavirus reported in surveillance aligns with model assumptions on reduced contact rates from March 2020 onward and is consistent with an increase in population susceptibility. The short to long term impact of this increased susceptibility places populations at risk of norovirus and rotavirus disease, but the scale of the impact remains uncertain and is subject to changes from the pandemic. While our analysis focuses on norovirus and rotavirus, the indirect impact of NPIs on disease incidence likely extends to other pathogens, and further modeling studies are needed to facilitate public health preparedness for potential resurgences. Overall, the study highlights the importance of continued investment to maintain robust national surveillance systems, which will remain critical to enable measures to limit the impact of these resurgences and provide essential information to public health organizations to implement preventive actions.

## Supplementary Information

**Additional file 1: Supplementary Figure 1.** Norovirus (left) and rotavirus (right) compartmental model structure. **Supplementary Figure 2.** Mobility estimates as the reduction in mobility on a scale from 0 (no mobility reduction) to 1 (100% mobility reduction) from February 4th, 2020. 

## Data Availability

For access to the norovirus and rotavirus data used in the study and code, applicants should submit a data request to Centers for Disease Control and Prevention’s National Outbreak Reporting System or National Respiratory and Enteric Virus Surveillance System.

## Abbreviations

NPI: Non-pharmaceutical interventions
CDC: Centers for Disease Control and Prevention
NORS: National Outbreak Reporting System
NoroSTAT: Norovirus Sentinel Testing and Tracking Network
NREVSS: National Respiratory and Enteric Virus Surveillance System
SIRS: Susceptible-infectious-recovered-susceptible
SIRV: Susceptible-infectious-recovered-vaccinated
US: United States
