# Supplementary material for: Predicting norovirus and rotavirus resurgence in the United States following the COVID-19 pandemic: a mathematical modelling study
Source: BMC Infect Dis. 2023 Apr 20;23:254. doi: 10.1186/s12879-023-08224-w (PMC10117239; doi:10.1186/s12879-023-08224-w)
Supplement: Supplementary file 1 — Additional file 1: Supplementary Figure 1. Norovirus (left) and rotavirus (right) compartmental model structure. Supplementary Figure 2. Mobility estimates as the reduction in mobility on a scale from 0 (no mobility reduction) to 1 (100% mobility reduction) from February 4th, 2020. [file 12879_2023_8224_MOESM1_ESM.docx]

**Supplemental Appendix**

**Supplemental Figure 1.** Norovirus (left) and rotavirus (right) compartmental model structure.

**
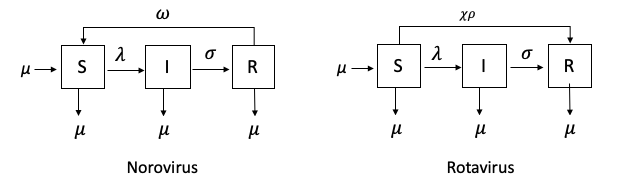
**

| **Norovirus Model Equations:**  $\frac{ds}{dt}=-\lambda(t)S+ \omega R\text{µ}N -\mu S$  $\frac{dI}{dt}=\lambda(t)S- \sigma I-\mu I$  $\frac{dR}{dt}=\sigma I-\omega R-\mu R$ | **Rotavirus Model Equations**  $\frac{ds}{dt}=-\lambda(t)S -\chi\rho S + \text{µ}N -\mu S$  $\frac{dI}{dt}=\lambda(t)S- \sigma I-\mu I$  $\frac{dR}{dt}=\sigma I+\chi\rho S-\mu R$ |
| --- | --- |

**µ = rate of birth and mortality**

**𝜌 = proportion vaccinated (rotavirus only)**

**𝜒 = vaccine efficacy (rotavirus only)**

**𝜎 = rate of loss of infection**

**𝜔 = rate of loss of immunity (norovirus only)**

**𝜆𝑖 = force of infection; rate at which susceptible individuals become infected**

**The force of infection was calculated as:**

$$\boldsymbol{\lambda=}\boldsymbol{\beta}\left( \boldsymbol{t} \right)\boldsymbol{*c(}\frac{\boldsymbol{I}}{\boldsymbol{N}}\boldsymbol{)}$$

**We modeled seasonal variation of norovirus and rotavirus transmission** $\boldsymbol{\beta(t)}$**:**

$$\boldsymbol{\beta(t)=q(1+A}\mathbf{cos}\boldsymbol{(2}\boldsymbol{\pi t + ϴ))}$$

**where** $\boldsymbol{q}$ **represents probability of transmission per contact for an individual,** $\boldsymbol{A}$**is the amplitude of the seasonal fluctuation and** $\boldsymbol{ϴ}$ **is the phase angle in years** $\boldsymbol{(t)}$**.**


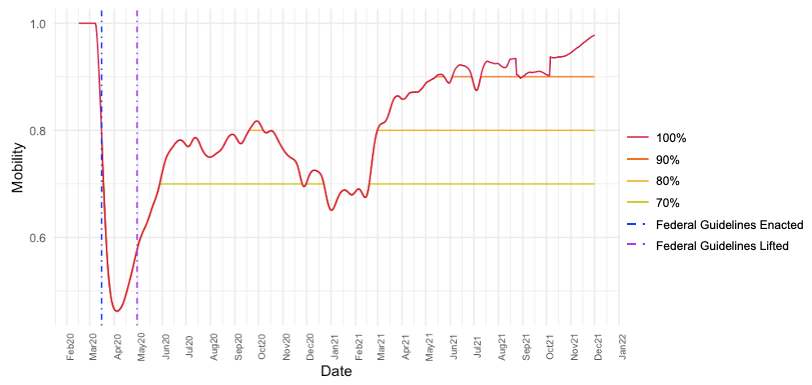


**Supplemental Figure 2.** Mobility estimates as the reduction in mobility on a scale from 0 (no mobility reduction) to 1 (100% mobility reduction) from February 4th, 2020. After April 5th, at which the maximum percent reduction of 53% reached, the mobility reduction was multiplied by the baseline number of contacts per individual. After that maximum percent reduction, we only increased contacts from that point to their scenario cap in which adults returned to 70%, 80%, 90%, or 100% (“return to normal”) of their pre-pandemic contact rates.
